# Predicting the prevalence of chronic kidney disease in the English population: a cross-sectional study

**DOI:** 10.1186/1471-2369-14-49

**Published:** 2013-02-25

**Authors:** Benjamin Kearns, Hugh Gallagher, Simon de Lusignan

**Affiliations:** 1 School of Health and Related Research, University of Sheffield, Sheffield S1 4DA, UK; 2 Division of Public Health Sciences and Education, St George’s-University of London, London, SW17 0RE, UK; 3 Department of Health Care Management and Policy, University of Surrey, Guildford, GU2 7XH, UK

**Keywords:** Chronic kidney disease, Renal disease, Prevalence, Statistical modelling, Association, Cardiovascular disease

## Abstract

**Background:**

There is concern that not all cases of chronic kidney disease (CKD) are known to general practitioners, leading to an underestimate of its true prevalence. We carried out this study to develop a model to predict the prevalence of CKD using a large English primary care dataset which includes previously undiagnosed cases of CKD.

**Methods:**

Cross-sectional analysis of data from the Quality Improvement in CKD trial, a representative sample of 743 935 adults in England aged 18 and over. We created multivariable logistic regression models to identify important predictive factors.

**Results:**

A prevalence of 6.76% was recorded in our sample, compared to a national prevalence of 4.3%. Increasing age, female gender and cardiovascular disease were associated with a significantly increased prevalence of CKD (p < 0.001 for all). Age had a complex association with CKD. Cardiovascular disease was a stronger predictive factor in younger than in older patients. For example, hypertension has an odds ratio of 2.02 amongst patients above average and an odds ratio of 3.91 amongst patients below average age.

**Conclusion:**

In England many cases of CKD remain undiagnosed. It is possible to use the results of this study to identify areas with high levels of undiagnosed CKD and groups at particular risk of having CKD.

**Trial registration:**

Current Controlled Trials ISRCTN: ISRCTN56023731. Note that this study reports the results of a cross-sectional analysis of data from this trial.

## Background

Chronic Kidney Disease (CKD) is largely asymptomatic [1]. Early identification affords opportunities to prevent and delay disease progression [2]. The potential benefits of active management include: reducing mortality and morbidity from cardiovascular diseases; progression to renal failure amongst patients with proteinuric disease; improving the quality of life for patients with more severe symptomatic disease; and reducing the use of resources and costs for health services [1].

Within England, CKD is included in a national pay-for-performance (P4P) scheme for chronic disease management. However, identification of CKD relies on opportunistic testing, and there is evidence that not everyone with CKD is being identified through the P4P scheme. Data from the Health Survey for England quote a national prevalence of 6% [3], the corresponding estimate from the P4P scheme is 4.3% [4]. Because of this difference, modelled estimates of the prevalence of CKD are required to support case-finding for CKD. These would enable public-health practitioners to identify and target areas where there is an under-detection of CKD and hence a need to promote awareness of its importance and improve existing local methods for identifying individuals at risk of CKD such as testing based on currently recommended risk factors [1].

There are existing models that may be used to estimate the prevalence of CKD for an area [5-8]. However, these models all have limitations: none of them use data from English patients and none check for interactions between variables.

We carried out this study to create a prevalence model for CKD. We used routinely collected data and a novel method to identify patients with CKD that has not been identified under the P4P scheme [9].

## Methods

### Data

The Quality Improvement in CKD (QICKD - ISRCTN56023731) trial [10,11] population includes a representative large sample of patients with CKD stages 3 to 5 in England [12]. The primary aim of the QICKD trial is to compare quality improvement interventions aimed at lowering systolic blood pressure in patients with CKD in primary care; ethical approval has been given for secondary analyses of the data. Ethics approval was received from the Oxford Research Ethics Committee (Committee C) (ref: 07/H0606/141). Here, the QICKD dataset was used to model the association between the prevalence of CKD and its potentially explanatory factors. This dataset contained patient-level data, extracted from the computer systems of 129 English general practices (GP), based in London, Surrey, Leicester, Birmingham, Cambridge and Sussex. The full dataset contained information on 930,997 people, of whom 743,935 are aged 18 or over. We only included people aged 18 or over to be consistent with the P4P scheme. The data are cross-sectional, extracted in 2009.

Cases of CKD stages 3 to 5 were strictly defined in accordance with the 2002 K-DOQI classification [13] on the basis of an estimated glomerular filtration rate (eGFR) of less than 60 ml/min/1.73 m^2^ for at least 90 days. Laboratories in England report eGFR using the four-variable modified diet in renal disease formula [14], with correction factors applied under the guidance of the National External Quality Assessment Service [15] to account for differences in local creatinine assays. People without a serum creatinine measurement in their electronic record were assumed for the purposes of the analysis not to have CKD.

We considered 11 potentially explanatory variables. These may be loosely classified as socio-demographic variables and variables about the presence or absence of cardiovascular disease. The socio-demographic variables were: age of subject (in years), gender, ethnicity, smoking status and deprivation score. Ethnicity was based on the 2001 England and Wales Census ‘5 + 1’ categories: ‘Asian’, ‘Black’, ‘Mixed’, ‘White’ ‘Other’, and ‘Not Stated’ [16]. There were two additional categories: ‘Not recorded’ occurred when there was an explicit code stating that ethnicity was not recorded, and ‘Missing’ was for missing ethnicity data. Smoking status was recorded as ‘never smoked’, ‘ex-smoker’, ‘smoker’ or it may be missing Ethnicity and smoking status were both modelled using dummy variables. Deprivation score was a continuous variable from the 2007 index of multiple deprivation [17], and based on the patient’s postcode [18].

The cardiovascular diseases considered were: diabetes, ischaemic heart disease (IHD), heart failure, hypertension, peripheral vascular disease (PVD) and stroke. These were modelled using dichotomous indicators. Data on systolic and diastolic blood pressure were available, but were not used due to high levels of missing data (23% of values were missing).

### Statistical analyses

Multivariable logistic regression was used to model the dependency of having CKD on the potentially explanatory factors. Model building was mainly based on the recommendations of Hosmer and Lemeshow [19]. Briefly, variables that showed a significant univariate association with CKD were included in a multivariable model. Manual backwards elimination was then applied; dropping non-significant variables one at a time. When no more variables could be deleted a check was made to see if any variables could be included. This gave a preliminary main-effects model. We checked if transformations were required for any continuous variables in this model, and considered possible interactions. We only considered interactions with age as this was known to be a strong predictor of CKD prevalence [5-7] and important interactions with age are often identified [20]. We used sample splitting to validate this approach; the sample was randomly split into two sub-sets (of approximately equal size), and the model-building process applied to both sub-sets.

Due to the large sample size nearly all variables were statistically significant with p-values less than 0.001. Instead we used clinical significance; for our study a variable was defined as clinically significant if its odds ratio (OR) was either above 1.49 or below 0.67. These values are derived from published CKD guidelines [1], in which it is stated that a rise in serum creatinine of over 20% should be considered significant. We checked for interactions by plotting the prevalence of CKD against age and stratifying by the levels of each factor (deprivation was categorised into quintiles). We observed a non-linear interaction between age and the cardiovascular diseases. This was modelled by introducing a dummy variable ‘below age 50’ which takes the value 0 if patients are below the age of 50 years and 1 otherwise, and including its interaction with the cardiovascular diseases.

We constructed a ‘clinical’ model (considering all the potentially explanatory variables) and a ‘parsimonious’ model which considered just age and gender as it was noted that sometimes these are the only variables for which data are available. We also present the results from the full main-effects model, as the use of this is sometimes recommended in the literature [20,21]. We used STATA version 10.1 [22] for all analyses.

Missing values for smoking status and ethnicity were treated as separate categories. Individuals with missing blood pressure readings (23%) were assumed not to have hypertension. Missing data for deprivation (19%) were due to a computer error during data collection, and so are assumed to be missing completely at random and were imputed by using a single implementation of the ICE procedure [23]. There were no missing data for any of the other variables.

Models were compared based on both their Akaike’s and the Bayesian information criteria (AIC and BIC respectively) [24], models with lower values were preferred. We performed a residual analysis using the deviance residuals to check goodness of fit. The Hosmer-Lemeshow test [19] may also be used to formally check goodness of fit. However, this measure is known to be of limited use for large sample sizes [25], so a graphical alternative was used: predicted and observed CKD prevalence were compared using deciles of predicted values [26,27]. The ability of the models to correctly classify patients was summarised by their ‘area under the receiver-operating-characteristic curve’ (AUROC) [28], along with their sensitivity, specificity, positive predictive value and negative predictive value. These values are known to be biased (give values that are too optimistic about model performance) when calculated based on the same data to which the model was built. To avoid this, we used a model built on one sub-set of the data, and calculated the statistics on the remaining sub-set.

### Additional analyses

The model-building process was repeated separately for CKD that had and had not been identified under the P4P scheme. Descriptive statistics were also produced for these two CKD classifications.

## Results

Of the 743 935 patients, 50 321 had CKD, giving a prevalence in the adult population of 6.76%. The mean age of the population was 46.7 years.

Variations in the prevalence of CKD were observed for all of the potentially explanatory factors (Table 1). With the exception of gender and the missing levels of both smoking status and ethnicity, all the univariable odds ratios are shrunk towards unity when controlled for differences in age. This shrinkage is the most notable for the cardiovascular diseases, for example the univariable odds ratio for heart failure changes from 16.07 to 2.78 after controlling for age.

**Table 1:** Summary statistics of co-variables used in the analysis

**Covariate**	**Sample Count (%)**	**Percent with CKD**	Odds ratios*		
			**Uni-variable**	**Bi-variable**	**Multi-variable**
**Gender**					
Female	373 929 (50%)	9.12%	1	1	1
Male	370 006 (50%)	4.38%	0.46	0.52	0.48
**Ethnicity**					
Asian	47 439 (6%)	4.31%	0.47	0.80	0.74
Black	34 497 (5%)	3.69%	0.40	0.82	0.77
Mixed	7873 (1%)	4.03%	0.43	**1.22**	**1.17**
White	224 806 (30%)	8.82%	1	1	1
Other	12 547 (2%)	2.17%	0.23	0.64	0.69
Not Recorded	8844 (1%)	8.44%	**0.95**	**0.96**	**1.09**
Not Stated	14 780 (2%)	9.53%	**1.09**	1.70	1.71
Missing	393 149 (53%)	6.21%	0.68	0.73	0.97
**Smoking status**					
Never smoked	357 588 (48%)	7.79%	1	1	1
Ex-smoker	153 051 (21%)	10.93%	1.45	**0.970**	**1.04**
Smoker	146 608 (20%)	3.69%	0.45	0.74	0.84
Missing	86 688 (12%)	0.36%	0.04	0.04	0.06
**Diabetes**					
No	708 072 (95%)	5.97%	1	1	1
Yes	35 863 (5%)	22.54%	4.59	1.82	1.47
**Stroke**					
No	728 836 (98%)	6.20%	1	1	1
Yes	15 099 (2%)	34.17%	7.86	1.59	1.27
**Heart Failure**					
No	738 669 (99%)	6.44%	1	1	1
Yes	5266 (1%)	52.51%	16.07	2.78	2.15
**Hypertension**					
No	635 309 (85%)	3.58%	1	1	1
Yes	108 626 (15%)	25.37%	9.15	2.36	1.87
**Ischaemic Heart Disease**					
No	717 929 (97%)	5.80%	1	1	1
Yes	26 006 (4%)	33.35%	8.13	2.77	1.49
**Peripheral Vascular Disease**					
No	738 875 (99%)	6.56%	1	1	1
Yes	5 060 (1%)	36.30%	8.12	1.86	1.44
	**Mean**	**St. Dev**			
**Age**	46.72	18.22	2.51	2.3 to 2.5	2.20
**Deprivation**	18.36	12.80	0.88	0.96	0.92

* Bivariable odds ratios are from the models including age. Multivariable odds ratios are from the model including all the predictors (constant term, coefficient: -3.36, 95% CI −3.39 to −3.33). Odds ratios for age are per 10 year increase, for deprivation they are per 10 point increase. Odds ratios in bold indicate values with a p-value > 0.001.

To reach the clinical model we applied the manual stepwise method, with the following exception:

It was not possible to apply clinical significance to multi-categorical variables (ethnicity and smoking status). These variables only had one significant level which related to missing data. After examining patterns of missing data, it was decided that smoking (but not ethnicity) data were not missing at random. Briefly, individuals with missing smoking status had a very low recorded prevalence for CKD, all cardiovascular diseases, and were also more likely to have a missing ethnicity. This suggests that the observed prevalence amongst subjects with missing smoking status is biased downwards, possibly due to a lack of GP contact. The remaining smoking categories were not clinically significant, and so smoking status was dropped.

Using the sample splitting approach, the final model in both sub-sets was the same. Hence these were pooled, and the model re-estimated based on all of the data. Summary measures of classification for the model are based on the sample-splitting approach. For the clinical model the variables deprivation score, PVD, stroke and smoking status were excluded.

**Figure 1:**
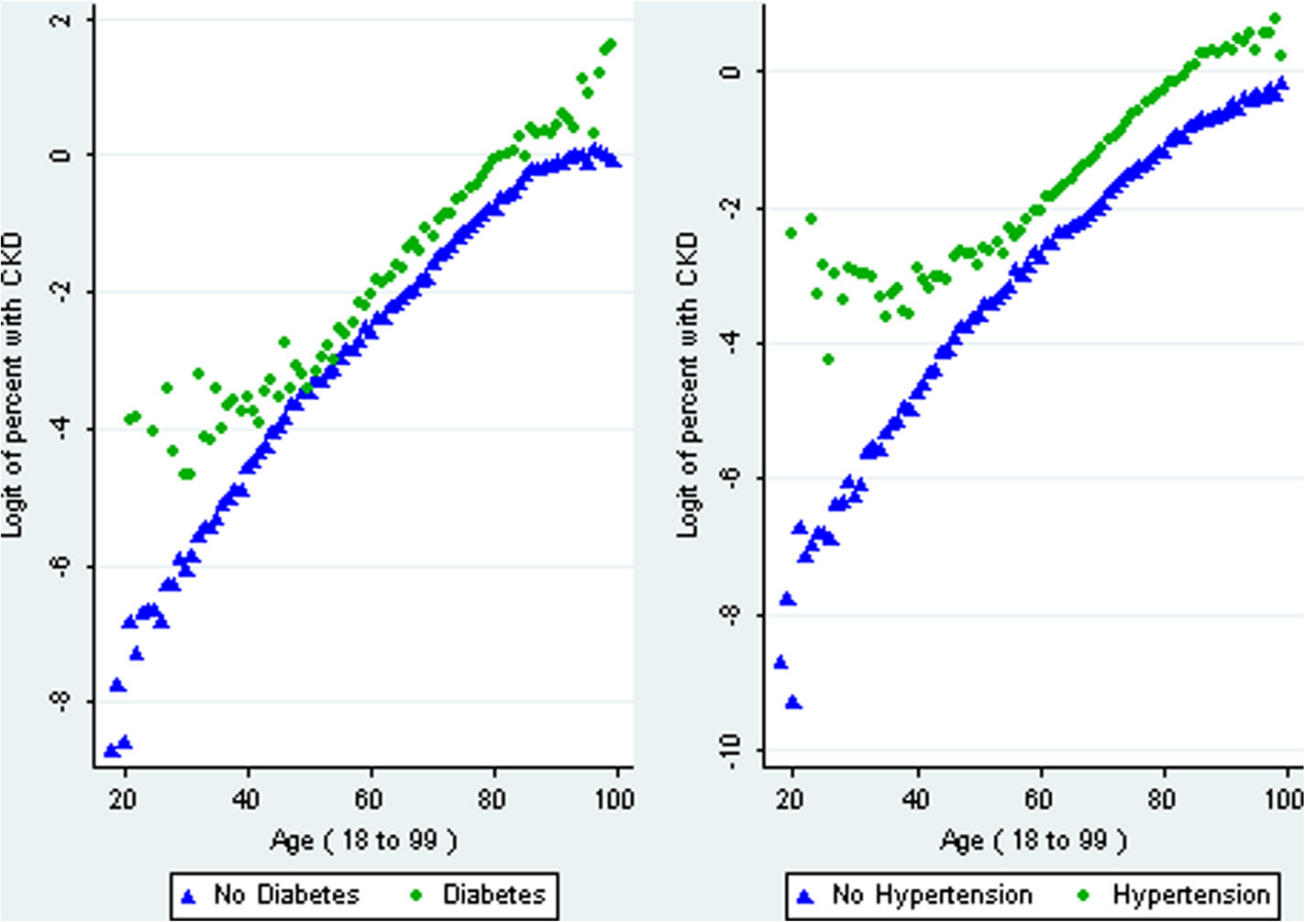
**Observed prevalence of CKD by age.** Results are stratified by diabetes status (left-hand pane) and hypertension status (right-hand pane).

A graphical check of the functional form for age [19] indicated that a quadratic term was required. This was confirmed by residual analysis and was also noticeable in the graphs constructed to check for interactions with age (Figure 1). Including the quadratic also reduced both information criteria.

For all the cardiovascular diseases an interaction with age was observed which appears to begin at the same age, two examples are shown in Figure 1. Because of these consistencies, an interaction with age was included for every cardiovascular disease in the final clinical model even though this interaction is only clinically significant for hypertension, and is not statistically significant for IHD and heart failure. The lack of statistical significance is likely to be due to the small numbers of people with the disease who are below average age.

Results for the clinical and the parsimonious model are presented in Table 2. Increasing age, female gender and white ethnicity were associated with a significantly increased prevalence of CKD, as was the presence of a cardiovascular disease. These cardiovascular diseases were stronger predictive factors in younger than in older patients. For example, using the results from the clinical model, the increases in the odds of CKD due to having hypertension is 2.02 amongst patients aged over 50 and 3.91 amongst patients aged below 50. Heart failure is associated with odds of 2.31 in older subjects and 3.14 amongst younger patients.

**Table 2:** Final multivariable logistic regression model for chronic kidney disease

	**Clinical**	**Parsimonious**	**Clinical**
	**Odds ratio (95% Confidence Interval)**		P-value**
**Age**			
per 10 years	2.93 (2.85 to 3.02)	3.11 (3.05 to 3.16)	<0.001
**Age**^**2**^			
per 10 years	0.99 (0.99 to 0.99)	0.99 (0.99 to 1.00)	<0.001
**Gender**			
Female*	1	1	
Male	0.48 (0.47 to 0.49)	0.52 (0.51 to 0.53)	<0.001
**Ethnicity**			
Asian	0.74 (0.70 to 0.78)		<0.001
Black	0.72 (0.68 to 0.77)		<0.001
Mixed	1.15 (1.01 to 1.31)		0.035
White	1		
Other	0.70 (0.61 to 0.80)		<0.001
Not Recorded	1.07 (0.98 to 1.16)		0.157
Not Stated	1.67 (1.56 to 1.79)		<0.001
Missing	0.85 (0.83 to 0.87)		<0.001
**Age < 50**			
No*	1		
Yes	1.11 (1.04 to 1.18)		0.002
**Heart Failure**			
No*	1		
Yes	2.37 (2.23 to 2.53)		<0.001
Yes and <50	1.34 (0.63 to 2.85)		0.45
**Hypertension**			
No*	1		
Yes	2.09 (2.05 to 2.14)		<0.001
Yes and <50	1.75 (1.59 to 1.92)		<0.001
**Ischaemic Heart Disease**			
No*	1		
Yes	1.67 (1.61 to 1.72)		<0.001
Yes and <50	1.14 (0.81 to 1.60)		0.45
**Constant**			
(Coeffeicient)	−3.63 (−3.67 to −3.58)	−3.56 (−3.58 to −3.53)	<0.001

*Baseline category for odds ratios. Both models fit to 743 935 individuals.

**Wald-based. P-values for age, age^2^, gender and the constant in the parsimonious models are the same.

**Figure 2:**
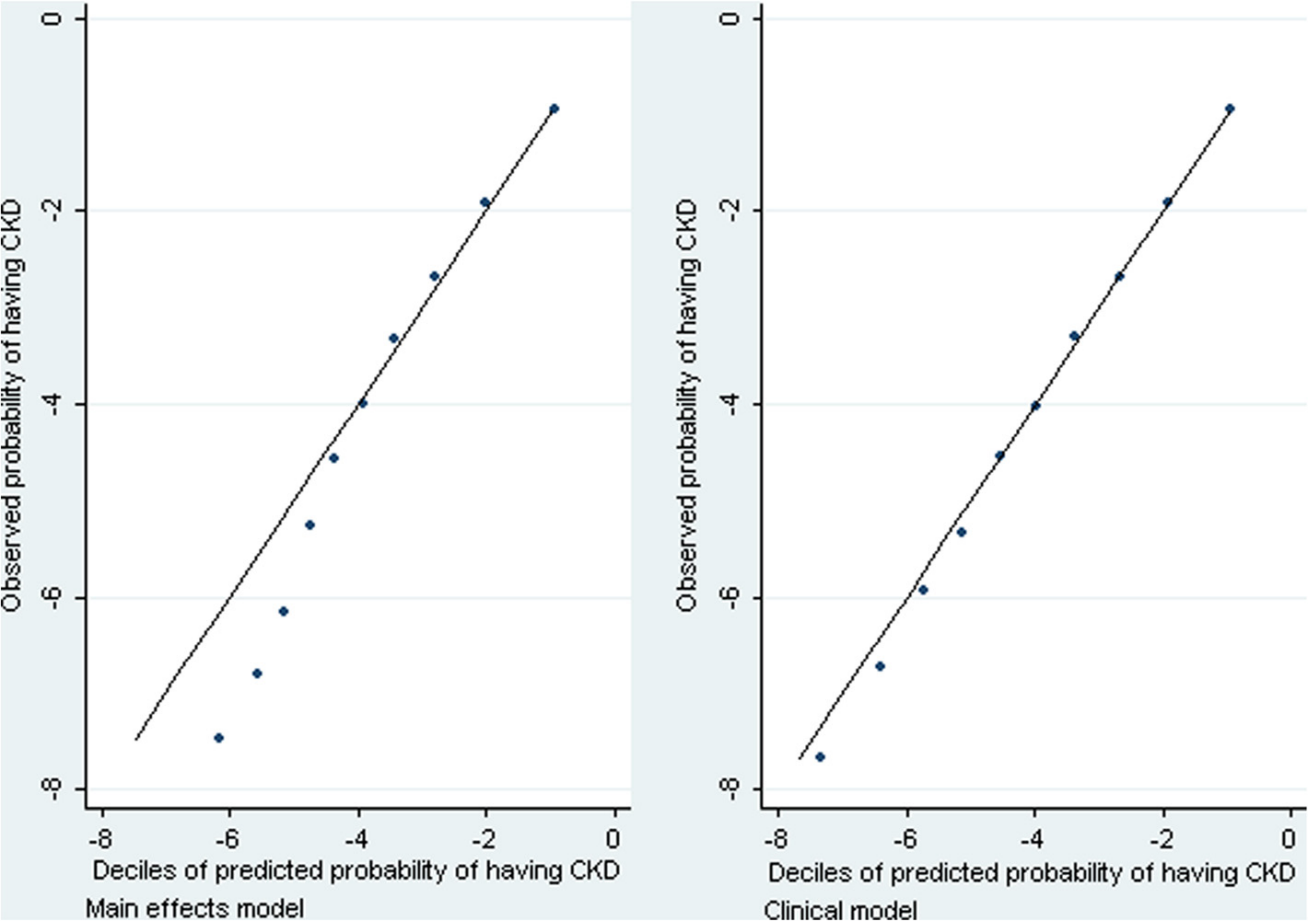
**Comparison of observed and expected probabilities of having CKD, plotted on the log-scale.** Results are presented for the full model (left-hand pane) and the clinical model (right-hand pane).

Summary measures and graphs for the three models suggested that they all fit the data well. There was very little difference between the in-sample classification measures, and the out-of sample measures. This suggests that the optimism due to building and evaluating a model on the same data is almost neglible for this analysis; possibly due to the large sample size. Out-of-sample AUROC scores were 0.898 (full model), 0.898 (clinical model) and 0.889 (parsimonious model) (Table 3). Graphs comparing observed and expected deciles of risk were similar for all three models, only those for the full and clinical model are shown (Figure 2). These graphs have been plotted on a log-scale, and show that use of the full model systematically underestimates CKD prevalence for this with low prevalence. This bias is mostly removed by the use of the clinical model, suggesting that it is due to the omission (in the full model) of the interactions between age and cardiovascular diseases.

**Table 3:** Summary measures of the regression models considered

**Model/Statistic**	**Main-effects**	**Clinical**	**Parsimonious**	**Clinical; no age**^**2**^
**AIC**	248 207	247 771	256 757	248 246
**BIC**	248 403	248 001	256 803	248 465
**DoF**	17	18	4	19
**In-sample classification measures**				
**Sensitivity**	22.05%	17.79%	10.78%	18.91%
**Specificity**	98.77%	99.07%	99.24%	98.97%
**AUROC**	0.899	0.899	0.890	0.899
**PPV**	58.07%	58.29%	50.98%	57.21%
**NPV**	94.27%	94.30%	93.85%	94.36%
**Out-of-sample classification measures**				
**Sensitivity**	22.15%	17.76%	10.94%	18.86%
**Specificity**	98.74%	99.06%	99.24%	98.96%
**AUROC**	0.898	0.898	0.889	0.898
**PPV**	57.23%	57.75%	50.88%	56.76%
**NPV**	94.33%	94.35%	93.91%	94.41%

AIC: Akaike’s Information criteria. BIC: Bayesian information criteria. DoF: Degrees of Freedom. AUROC: Area under the receiver operating characteristic curve. PPV: Positive predictive value. NPV: Negative predictive value.

Applying the model-building process to just patients with identified CKD gave similar results to using all cases of CKD, the main difference was the inclusion of diabetes. However, when it was applied to patients with unidentified CKD the resulting model was very different. None of the cardiovascular diseases were clinically significant predictors, whilst being of an Asian or Black ethnicity was a much stronger predictor of not having CKD (Additional file 1 and Additional file 2). More research is required into why these differences arise.

## Discussion

We have developed new models to give accurate predictions of CKD. Increasing age, female gender, white ethnicity and cardiovascular disease were all associated with an increased prevalence of CKD. In addition, we have also shown that there is a complex association with age which in turn interacts with cardiovascular disease. The effects of these diseases were greater amongst younger than older adults. The pattern of this interaction was very similar for all the cardiovascular diseases.

The results of our study support those previously published by confirming the important roles of age, gender [5-8,29,30] and CVD [31-33] in predicting cases of CKD. We also found statistically significant associations with deprivation and ethnicity, but these were not clinically significant; this may explain why there is weak or mixed evidence on their importance in predicting the prevalence of CKD [34].

There are four studies that look at multivariable models for predicting the prevalence of CKD [5-8]. ORs for female varied between 1.19 and 1.49. All four studies considered the effect of hypertension and diabetes; for the ORs ranged from 1.4 to 1.72, whilst for the latter ORs ranged from 0.9 to 2.68. None of the studies considered interactions between the cardiovascular diseases and age.

Both Bang *et al*. [5] and Whaley-Connell *et al*. [7] considered ethnicity. Bang *et al*. [5] found that, compared to non-Whites, Whites had a statistically significant univariable odds ratio (2.1, p = 0.03) of having CKD, but that ethnicity was not a significant predictor in the multivariable model. Whaley-Connell *et al*. [7] considered two different cohorts; in one White ethnicity was associated with a statistically significant odds ratio of 1.23 (p < 0.001), in the other it had a non-significant odds ratio of 0.91 (p = 0.2).

All four studies confirm the important effect of age; Chadban *et al*. [6] compared subjects aged under 65 to those aged over 65 and reported an odds ratio of 102 (p < 0.001). The other three studies categorised age, and reported significant odds ratios for all categories. Our study further shows that age has a complex association with CKD.

The results of this model are also consistent with cohort studies of CKD in showing that there are interactions between CKD, age and cardiovascular disease [35]. Other interactions between age and cardiovascular disease have also been reported in the literature [20,36].

This is the first study that we are aware of that provides multivariable models for predicting the prevalence of CKD in England. Our results are similar to those based in other countries in identifying important variables, but the magnitude of the associations often vary. For example, we found an odds ratio for female gender of about two for all three models; a larger value than that reported in the other studies.

We have identified important interactions between age and cardiovascular disease in predicting the prevalence of CKD. These interaction have not been included in any of the existing models for predicting the prevalence of CKD (or in models for predicting the incidence of CKD [29,30]) despite evidence of its importance in the literature. Service planning based on existing models, which fail to capture these interactions, may result in a mismatch between supply and demand for renal services in primary and secondary care. More accurate predictions of CKD prevalence may allow more accurate targeting of resources toward areas of unmet need. A particular strength of our study is the large sample size available. This resulted in increased power to estimate coefficients, especially for interactions. The large sample size, along with the consistency of findings when employing sample-splitting, suggest that the interactions identified in this study will generalise to the rest of the England CKD population.

The QICKD study includes patients whose CKD has not been diagnosed in general practice, and so these estimates may be compared with the P4P CKD indicator to determine areas with high levels of un-met need. At a national level, the P4P indicator in England gives a prevalence of CKD of 4.3% [4], we reported a prevalence of 6.76%, suggesting that over a third of people with CKD are not known to their GP. This confirms findings in the recent Health Survey for England, which also included cases of CKD not diagnosed in general and reported a prevalence of 6% [3]. Analysis of patients with unidentified CKD suggests that their risk profile may be different to patients with identified CKD, this is an area that requires further research.

### Limitations

Using cross-sectional data is a limitation, as it is known that rates of progression vary by patient characteristics [1]. The results of this analysis may be used to identify areas with a high prevalence of CKD, where early identification will be beneficial in reducing both progression to renal failure and morbidity from cardiovascular disease. However, when targeting resources for CKD, consideration should also be given to variations in rates of progression across populations. We also made no distinction between varying levels of kidney disease. The available literature suggests that the risk profile for CKD may vary as kidney disease progresses; for example it has been shown that the proportion of males with CKD increases with worsening stage [37], and a recent study found that non-white ethnicity was a significant predictor of renal replacement therapy [38]. As renal failure can be devastating for the patient and very expensive [1], more research is required into rates of progression.

We have assumed that people without a serum creatinine measurement did not have CKD. Whilst this is consistent with previous approaches [39], there was no measurement recorded for 56% of the sample. Hence the prevalence of CKD reported here is likely to be an under-estimate.

The choice to use clinical significance instead of statistical significance posed some problems. In particular the choice of whether or not to include the multi-categorical variables ethnicity and smoking status was slightly arbitrary. The importance of all the omitted variables warrants further research. For the continuous variables the value of the odds ratio (and hence their clinical significance) depends on the units reported. We used the odds ratio per 10-year increase in age, which is commonly employed in the literature [5,8,29,30,34]. For deprivation we used a 10-point increase (deprivation values range between 0.75 and 77.37). Using the results from the full main-effects model, we would need to use a 45-point increase in deprivation for it to become clinically significant, and a 5-year increase in age for it to become not clinically significant.

We did not anticipate *a priori* the nature of the observed interactions between age and the cardiovascular diseases and this feature needs to be independently confirmed. In addition there is scope to improve the modelling of this interaction; noticeably the choice of at what age to start modelling the interaction warrants further research.

## Conclusions

CKD is largely asymptomatic, making accurate identification and subsequent management of patients at risk of progression difficult. However, identification is important because progression of patients to symptomatic disease impairs their quality of life and results in increased costs for health services. Although included within the P4P scheme, it is recognised that CKD is under-ascertained within primary care in England.

We have developed disease prevalence models for CKD that will allow decision makers to identify areas where the P4P rates are lower than expected and target these for possible public health interventions. The results of this study may also be used to identify sub-groups or patient profiles in whom the demands for renal services and treatment may be increased, such as young people with a cardiovascular disease.

## Abbreviations

AIC: Akaike’s information criteria; AUROC: Area under the receiver operating characteristic curve; BIC: Bayesian information criteria; CKD: Chronic kidney disease; DoF: Degrees of freedom; eGFR: Estimated glomerular filtration rate; IHD: Ischaemic heart disease; P4P: Pay for performance; PVD:Peripheral vascular disease; QICKD: Quality improvement in chronic kidney disease.

## Competing interests

There was no specific funding for this project. However, SdeL is the principal investigator for the QICKD trial, SdeL led the expert reference group that created the CKD Pay-for-Performance indicator; and is now a GP Advisor to NICE for CKD. SdeL has received funding to attend and present at two European conferences in the last five years, and Editorial fees for two articles (joint publications with HG).

## Authors’ contributions

BK performed the analysis and interpretation of the data for this manuscript and drafted the article. He had full access to all the data in the study and takes responsibility for the integrity of the data and the accuracy of the data analysis. SdeL and HG were involved in the conception of the study and acquisition of the data. They also critically revised the manuscript for important intellectual content, contributing to all drafts of the manuscript. SdeL also contributed to the analysis and interpretation of the data. All authors have given approval for the final version to be published.

## Pre-publication history

The pre-publication history for this paper can be accessed here:

http://www.biomedcentral.com/1471-2369/14/49/prepub

## Supplementary Material

Additional file 1 Summary statistics for the total sample, and for subjects with chronic kidney disease (CKD), broken-down by identified and unidentified CKD. Click here for file

Additional file 2 Full main-effects and ‘clinical’ multivariable logistic regression models for subjects with identified Chronic Kidney Disease. Click here for file
