# Supplementary material for: Predicting the prevalence of chronic kidney disease in the English population: a cross-sectional study
Source: BMC Nephrol. 2013 Feb 25;14:49. doi: 10.1186/1471-2369-14-49 (PMC3598334; doi:10.1186/1471-2369-14-49)
Supplement: Additional file 1 — Summary statistics for the total sample, and for subjects with chronic kidney disease (CKD), broken-down by identified and unidentified CKD. [file 1471-2369-14-49-S1.doc]

**Summary statistics for the total sample, and for subjects with chronic kidney disease (CKD), broken-down by identified and unidentified CKD**.

| **Covariate** | **Sample Count (%)** | | | |
| --- | --- | --- | --- | --- |
|  | **Total sample**  **N = 743 935** | **Identified CKD**  **N = 29 054** | | **Unidentified CKD**  **N = 21 267** |
| **Gender** | | | | |
| Female | 373 929 (50%) | | 18 773 (64.61%) | 15 335 (72.11%) |
| Male | 370 006 (50%) | | 10 281 (35.39%) | 5 932 (27.89%) |
| **Ethnicity** | | | | |
| Asian | 47 439 (6%) | | 1 570 (5.4%) | 476 (2.24%) |
| Black | 34 497 (5%) | | 1 118 (3.85%) | 155 (0.73%) |
| Mixed | 7873 (1%) | | 219 (0.75%) | 98 (0.46%) |
| White | 224 806 (30%) | | 13 617 (46.87%) | 6 220 (29.25%) |
| Other | 12 547 (2%) | | 152 (0.52%) | 120 (0.56%) |
| Not Recorded | 8844 (1%) | | 499 (1.72%) | 247 (1.16%) |
| Not Stated | 14 780 (2%) | | 805 (2.77%) | 603 (2.84%) |
| Missing | 393 149 (53%) | | 11 074 (38.12%) | 13 348 (62.76%) |
| **Smoking status** | | | | |
| Never smoked | 357 588 (48%) | | 15 859 (54.58%) | 12 007 (56.46%) |
| Ex-smoker | 153 051 (21%) | | 10 238 (35.24%) | 6 497 (30.55%) |
| Smoker | 146 608 (20%) | | 2 840 (9.77%) | 2 564 (12.06%) |
| Missing | 86 688 (12%) | | 117 (0.4%) | 199 (0.94%) |
| **Diabetes** | | | | |
| No | 708 072 (95%) | | 23 001 (79.17%) | 19 236 (90.45%) |
| Yes | 35 863 (5%) | | 6 053 (20.83%) | 2 031 (9.55%) |
| **Stroke** | | | | |
| No | 728 836 (98%) | | 25 391 (87.39%) | 19 771 (92.97%) |
| Yes | 15 099 (2%) | | 3 663 (12.61%) | 1 496 (7.03%) |
| **Heart Failure** | | | | |
| No | 738 669 (99%) | | 26 948 (92.75%) | 20 608 (96.9%) |
| Yes | 5266 (1%) | | 2 106 (7.25%) | 659 (3.1%) |
| **Hypertension** | | | | |
| No | 635 309 (85%) | | 9 971 (34.32%) | 12 791 (60.14%) |
| Yes | 108 626 (15%) | | 19 083 (65.68%) | 8 476 (39.86%) |
| **Ischaemic Heart Disease** | | | | |
| No | 717 929 (97%) | | 22 718 (78.19%) | 18 929 (89.01%) |
| Yes | 26 006 (4%) | | 6 336 (21.81%) | 2 338 (10.99%) |
| **Peripheral Vascular Disease** | | | | |
| No | 738 875 (99%) | | 27 720 (95.41%) | 20 764 (97.63%) |
| Yes | 5 060 (1%) | | 1 334 (4.59%) | 503 (2.37%) |
|  | **Mean (Std. Dev)** | | | |
| **Age** | 46.72 (18.22) | | 75.26 (12.28) | 68.58 (14.88) |
| **Deprivation** | 18.36 (12.80) | | 17.77 (13.00) | 13.71 (11.49) |
