# Supplementary material for: Predicting the prevalence of chronic kidney disease in the English population: a cross-sectional study
Source: BMC Nephrol. 2013 Feb 25;14:49. doi: 10.1186/1471-2369-14-49 (PMC3598334; doi:10.1186/1471-2369-14-49)
Supplement: Additional file 2 — Full main-effects and ‘clinical’ multivariable logistic regression models for subjects with identified Chronic Kidney Disease. [file 1471-2369-14-49-S2.doc]

**Full main-effects and ‘clinical’ multivariable logistic regression models for subjects with identified Chronic Kidney Disease**.

|  | **Full main-effects** | **Clinical** | **Clinical** |
| --- | --- | --- | --- |
|  | **Odds Ratio (95% Confidence Interval)** | | **P-value**** |
| **Age** |  |  |  |
| per 10 years | 2.30 (2.27 to 2.32) | 3.59 (3.42 to 3.77) | <0.001 |
| **Age^2^** |  |  |  |
| per 10 years | 1.00 (0.99 to 1.01) | 0.99 (0.99 to 0.99) | <0.001 |
| **Gender** |  |  |  |
| Female* | 1 | 1 |  |
| Male | 0.63 (0.61 to 0.65) | 0.63 (0.61 to 0.65) | <0.001 |
| **Ethnicity** |  |  |  |
| Asian | 0.83 (0.77 to 0.89) | 0.84 (0.79 to 0.90) | <0.001 |
| Black | 1.12 (1.03 to 1.21) | 1.04 (0.97 to 1.12) | 0.24 |
| Mixed | 1.24 (1.05 to 1.46) | 1.25 (1.07 to 1.47) | 0.004 |
| White | 1 | 1 |  |
| Other | 0.65 (0.53 to 0.80) | 0.66 (0.56 to 0.78) | <0.001 |
| Not Recorded | 1.14 (1.02 to 1.27) | 1.12 (1.01 to 1.24) | 0.34 |
| Not Stated | 1.29 (1.18 to 1.42) | 1.28 (1.17 to 1.39) | <0.001 |
| Missing | 0.62 (0.60 to 0.64) | 0.57 (0.55 to 0.58) | <0.001 |
| **Smoking status** |  |  |  |
| Never smoked | 1 |  |  |
| Ex-smoker | 1.07 (1.04 to 1.11) |  |  |
| Smoker | 0.86 (0.82 to 0.91) |  |  |
| Missing | 0.07 (0.05 to 0.08) |  |  |
| **Mean age** |  |  |  |
| 0* |  | 1 |  |
| 1 |  | 1.01 (0.90 to 1.13) | 0.855 |
| **Diabetes** |  |  |  |
| No* | 1 | 1 |  |
| Yes | 1.63 (1.56 to 1.69) | 1.58 (1.52 to 1.64) | <0.001 |
| x Mean age |  | 1.20 (0.97 to 1.48) | 0.099 |
| **Stroke** |  |  |  |
| No* | 1 |  |  |
| Yes | 1.27 (1.21 to 1.34) |  |  |
| **Heart Failure** |  |  |  |
| No* | 1 | 1 |  |
| Yes | 1.99 (1.85 to 2.13) | 2.16 (2.03 to 2.30) | <0.001 |
| x Mean age |  | 1.89 (0.77 to 4.62) | 0.163 |
| **Hypertension** |  |  |  |
| No* | 1 | 1 |  |
| Yes | 2.41 (2.34 to 2.49) | 2.5 (2.43 to 2.57) | <0.001 |
| x Mean age |  | 2.68 (2.34 to 3.07) | <0.001 |
| **Ischaemic Heart Disease** | |  |  |
| No* | 1 | 1 |  |
| Yes | 1.57 (1.51 to 1.64) | 1.66 (1.60 to 1.72) | <0.001 |
| x Mean age |  | 1.06 (0.68 to 1.65) | 0.789 |
| **Peripheral Vascular Disease** | |  |  |
| No* | 1 |  |  |
| Yes | 1.39 (1.29 to 1.51) |  |  |
| **Constant** |  |  |  |
| (Coeffeicient) | -4.37 (-4.42 to -4.32) | -4.78 (-4.85 to -4.71) | <0.001 |

**Full main-effects and ‘clinical’ multivariable logistic regression models for subjects with unidentified Chronic Kidney Disease.**

|  | **Full main-effects** | **Clinical** | **Clinical** |
| --- | --- | --- | --- |
|  | **Odds Ratio (95% Confidence Interval)** | | **P-value**** |
| **Age** |  |  |  |
| per 10 years | 1.82 (1.80 to 1.84) | 2.60 (2.55 to 2.65) | <0.001 |
| **Age^2^** |  |  |  |
| per 10 years | 0.82 (0.81 to 0.83) | 0.99 (0.99 to 0.99) | <0.001 |
| **Gender** |  |  |  |
| Female* | 1 | 1 |  |
| Male | 0.43 (0.42 to 0.45) | 0.42 (0.41 to 0.43) | <0.001 |
| **Ethnicity** |  |  |  |
| Asian | 0.61 (0.55 to 0.68) | 0.55 (0.50 to 0.60) | <0.001 |
| Black | 0.31 (0.26 to 0.38) | 0.27 (0.23 to 0.32) | <0.001 |
| Mixed | 1.00 (0.80 to 1.24) | 0.99 (0.81 to 1.22) | <0.936 |
| White | 1 | 1 |  |
| Other | 0.89 (0.72 to 1.09) | 0.82 (0.68 to 0.99) | <0.039 |
| Not Recorded | 0.99 (0.87 to 1.14) | 1.00 (0.88 to 1.15) | <0.941 |
| Not Stated | 2.09 (1.90 to 2.29) | 2.01 (1.83 to 2.19) | <0.001 |
| Missing | 1.55 (1.50 to 1.61) | 1.39 (1.35 to 1.44) | <0.001 |
| **Smoking status** |  |  |  |
| Never smoked | 1 |  |  |
| Ex-smoker | 0.98 (0.94 to 1.01) |  |  |
| Smoker | 0.88 (0.83 to 0.92) |  |  |
| Missing | 0.07 (0.06 to 0.09) |  |  |
| **Diabetes** |  |  |  |
| No* | 1 |  |  |
| Yes | 1.04 (0.98 to 1.10) |  |  |
| **Stroke** |  |  |  |
| No* | 1 |  |  |
| Yes | 0.96 (0.90 to 1.02) |  |  |
| **Heart Failure** |  |  |  |
| No* | 1 |  |  |
| Yes | 1.03 (0.93 to 1.13) |  |  |
| **Hypertension** |  |  |  |
| No* | 1 |  |  |
| Yes | 1.11 (1.07 to 1.15) |  |  |
| **Ischaemic Heart Disease** | |  |  |
| No* | 1 |  |  |
| Yes | 1.05 (0.99 to 1.11) |  |  |
| **Peripheral Vascular Disease** | |  |  |
| No* | 1 |  |  |
| Yes | 1.18 (1.05 to 1.31) |  |  |
| **Constant** |  |  |  |
| (Coeffeicient) | -3.63 (-3.67 to -3.58) | -4.03 (-4.07 to -4.00) | <0.001 |
